# Parallel group independent component analysis for massive fMRI data sets

**DOI:** 10.1371/journal.pone.0173496

**Published:** 2017-03-09

**Authors:** Shaojie Chen, Lei Huang, Huitong Qiu, Mary Beth Nebel, Stewart H. Mostofsky, James J. Pekar, Martin A. Lindquist, Ani Eloyan, Brian S. Caffo

**Affiliations:** 1 Department of Biostatistics, Johns Hopkins University, Baltimore, United States of America; 2 School of Medicine, Johns Hopkins University, Baltimore, United States of America; 3 Kennedy Krieger Institute, Baltimore, United States of America; 4 Department of Radiology and Radiological Science, Johns Hopkins University, Baltimore, United States of America; 5 Department of Neurology, Johns Hopkins University, Baltimore, United States of America; 6 Department of Biostatistics, Brown University, Providence, Rhode Island, United States of America; University of Texas at Austin, UNITED STATES

## Abstract

Independent component analysis (ICA) is widely used in the field of functional neuroimaging to decompose data into spatio-temporal patterns of co-activation. In particular, ICA has found wide usage in the analysis of resting state fMRI (rs-fMRI) data. Recently, a number of large-scale data sets have become publicly available that consist of rs-fMRI scans from thousands of subjects. As a result, efficient ICA algorithms that scale well to the increased number of subjects are required. To address this problem, we propose a two-stage likelihood-based algorithm for performing group ICA, which we denote Parallel Group Independent Component Analysis (PGICA). By utilizing the sequential nature of the algorithm and parallel computing techniques, we are able to efficiently analyze data sets from large numbers of subjects. We illustrate the efficacy of PGICA, which has been implemented in R and is freely available through the Comprehensive R Archive Network, through simulation studies and application to rs-fMRI data from two large multi-subject data sets, consisting of 301 and 779 subjects respectively.

## 1 Introduction

Independent component analysis (ICA) is a blind source separation technique [1] that assumes the observed signals are linear mixings of independent underlying sources. A framework for using ICA to make group inferences from functional Magnetic Resonance Imaging (fMRI) data was first introduced by [2]. A major methodological contribution of this work was the circumvention of the permutation ambiguity of ICA by eliminating the requirement to match components across subjects. Since its introduction, ICA has become an extremely popular approach to analyzing fMRI data, as it does not require the a priori definition of a hemodynamic response function or seed regions of interest and is able to capture both spatial and temporal inter-subject variability [3–7]. Several algorithms have been developed to estimate parameters in ICA [8, 9], but most existing algorithms require data to be concatenated across subjects and then reduced via principal component analysis to a set of spatial eigenvectors representative of the group. A single run of ICA is then performed on these group-level principal components after which subject-specific spatial maps (SMs) and time courses (TCs) are estimated using various back-projection techniques. At the group-level ICA step, different ICA algorithms such as Infomax and FastICA can be used to estimate group-level ICs. Infomax is the default setting in the widely used Group ICA toolbox (GIFT) toolbox due to its reliability [10]. Following the estimation of group-level ICs, a wide variety of methods can be used to then reconstruct subject-specific independent components, such as GICA 1, GICA 2, GICA 3, dual regression and Group Information Guided ICA (GIG-ICA). Both dual regression and GIG-ICA have great scalability [5–7]. However, concerns have recently been raised about the scalability of the (first step) group-level ICA methods [11]. With the neuroscience community taking cues from the the crowdsourcing model of labor and encouraging the public distribution of large collections of data including thousands of subjects collected at multiple sites, the development of algorithms for analyzing such high dimensional data is imperative.

A common starting point for most group ICA approaches is the principal component analysis (PCA), or the singular value decomposition (SVD). While the PCA/SVD is a means for avoiding the estimation of an overdetermined system, it is also the means for throwing away massive amounts of data through repeated application [11]. Scalable PCA/SVD algorithms are required to handle large data efficiently in group ICA. Multiple efficient methods have been proposed, such as the block-lanczos [12], Multi power iteration (MPOWIT) [13], small memory iterated group PCA (SMIG) and MELODIC’s incremental group PCA (MIGP) [11]. There are also three data reduction methods which can be used to obtain an approximate PCA subspace efficiently in GIFT [10].

A notable exception is the work by [14], which does not require repeated SVD steps to be scalable. Gaussian distributional assumptions can provide little insight to further explore the data, and we are motivated to search for components that are as non-Gaussian as possible. The densities of the underlying components in the algorithm proposed by [14] are approximated with finite mixtures of smooth densities, while the time courses for each subject are updated using a gradient-based optimization algorithm. A Quasi-Newton algorithm is used for optimization to estimate the parameters in the mixing matrix.

In this paper, we propose a more direct solution to the scalability issue described by [11] by building upon the two-stage likelihood-based algorithm proposed by [14] and use parallel computing techniques to improve algorithmic performance for large groups of observations. The algorithm proposed by [14], is scalable, but performs calculations serially. We decompose the problem into computationally unrelated tasks and then distribute them over a parallel computing system. The proposed Parallel Group Independent Component Analysis (PGICA) is different from fastICA and JADE in that the algorithm is likelihood-based and uses maximum likelihood estimation (MLE) for parameter estimation. Compared to the ML implementation of ICA by [15], PGICA does not require a highly restricted likelihood. Instead, flexible mixtures of Gaussian densities are used to approximate the densities of the underlying components. Another advantage of PGICA is its ability to analyze massive data. Current group ICA algorithms have limited power for scaling to analyze large data sets, especially in the field of resting state fMRI analysis because they require data to first be concatenated across subjects and reduced via PCA prior to estimation of group-level independent components. The current standard is thus to throw away massive amounts of data with repeated applications of the SVD [11]. PGICA can handle hundreds to thousands of subjects simultaneously with the help of parallel computing. Many parallel programming environments exist that provide basic tools, language features and application programming interfaces (APIs) needed to construct a parallel program. Widely used environments include: OpenMP (thread-level parallelization), MPI (cluster-level) and CUDA / OpenCL (GPGPU-level). The RSGE package in the R software provides an interface to perform cluster-level parallel programming on Sun Grid Engines (SGE) [16] and the SNOW package can be used for thread-level parallel computing [17]. In newer versions of R (≥2.14.0), the package *parallel* is included in its core, which provides drop-in replacements for most of the functionalities of *snow*. The R package we built for this work is based on package *parallel*. At the end, we illustrate the performance of PGICA by applying it to rs-fMRI data from two large multi-subject data sets. The first is a collection of 301 adults, while the second is a set of 779 fMRI scans, consisting of 379 with autism spectrum disorder (ASD) and 400 typically developing controls.

## 2 Materials and methods

### 2.1 The ICA model

A general term that indexes a broad class of models, ICA has several algorithmic implementations and theoretical foundations, but the linear factor analytic model with the assumption of independent underlying factors is the primary commonality of all ICA algorithms [18]. In this paper, we focus on noise-free ICA, a version of ICA which only requires an “unmixing” of the input data matrix. (Thus, the noise in the data is absorbed into the estimated independent components.)

Suppose that for each subject *i*, *i* = 1, …, *I*, a *T* × *V* dimensional matrix is observed. In the neuroimaging context, the rows represent time points and the columns represent voxels. Let **X**_*i*_(*t*, *v*) represent row *t*, column *v* of **X**_*i*_. (The same notational convention applies to other vectors and matrices.) The noise-free group ICA decomposition model can be expressed as follows.

$$\begin{array}{c} X_{i}\left(t,v\right)=\sum_{q=1}^{Q}A_{i}\left(t,q\right)S\left(q,v\right), \end{array}$$ (1)

for *i* = 1, …, *I*. This model assumes that the spatio-temporal process, *X*_*i*_(*t*, *v*), for each subject, *i*, can be decomposed into a finite sum of products between subject-specific time series, *A*_*i*_(*t*, *q*), and subject-independent spatial maps, *S*(*q*, *v*). Let $X={\left[X_{1}^{T}...X_{I}^{T}\right]}^{T}$ and $A={\left[A_{1}^{T}...A_{I}^{T}\right]}^{T}$ be the *IT* × *V* and *IT* × *Q* matrices obtained by stacking the **X**_**i**_ and **A**_**i**_ respectively, then the above model is equivalent to **X** = **AS**. In the fMRI context, one often interprets **S**(*q*, ⋅) as brain networks and **A**_*i*_ as subject specific temporal mixing matrices [2].

As a technical consideration, Eq (1) maybe overdetermined. So we first preprocess the data at subject level via an singular value decomposition (SVD) on the observed matrices and retains only the first *Q* components for each subject. This first-step dimension reduction on the temporal domain via SVD is unavoidable. After the first-step dimension reduction, the new *T* is generally a lot smaller than the original number of scans. Henceforth, we assume that the time points after dimension reduction is equal to the number of components to estimate, i.e. *T* = *Q*. The data are whitened before PGICA is applied, so the square matrices **A**_*i*_ are orthogonal and one can define their inverses as $W_{i}=A_{i}^{-1}$ and the densities of the underlying components as *f*_1_, …, *f*_*Q*_. Thus, for a given *q*, ${\left\{S\left(q,v\right)\right\}}_{v=1}^{V}$ can be considered as *V* iid draws from *f*_*q*_.

### 2.2 Parameter estimation

The likelihood of the above model can be written as

$$\begin{array}{c} L\left(W,f\right)=\prod_{i=1}^{I}\prod_{v=1}^{V}\prod_{q=1}^{Q}f_{q}(\sum_{l=1}^{Q}w_{iql}x_{ilv})\left|\text{det}\left(W_{i}\right)\right|, \end{array}$$ (2)

If the *f*_*q*_ were known, any optimization algorithm could be used to obtain the maximum likelihood estimation (MLE) of **W**_*i*_. However, since the densities of the underlying components are unknown, an iterative algorithm must be implemented that alternates between density estimation and estimation of the **W**_*i*_. This manuscript uses mixture density estimates (MDE) introduced by [19]. Specifically, we parameterize the densities as:

$$\begin{array}{c} f_{q}\left(s\right)=\sum_{j=1}^{J_{q}}\theta_{qj}\frac{1}{\sigma_{q}}\phi (\frac{s-\mu_{qj}}{\sigma_{q}}), \end{array}$$ (3)

where *ϕ*(⋅) is the standard normal density function. The number of densities in the mixture $J_{q}=1+\left\lfloor \frac{2}{3}{\text{Range}}_{v}\left\{S\left(q,v\right)\right\}\right\rfloor$ is chosen empirically. Note that although this *J*_*q*_ performs well in practice, there are other summary statistics (such as percentiles) that are more robust to the skewed distribution of **S**(*q*, *v*), which should be tried in later versions of the algorithm. Similarly, $\mu_{qj}={\text{min}}_{v}S\left(q,v\right)+\frac{j-1}{J_{q}-1}{\text{Range}}_{v}\left\{S\left(q,v\right)\right\}$ for *j* = 1, ⋯, *J*_*q*_. The underlying rationale behind this is to set the means *μ*_*qj*_ as an equally spaced grid between the extremes of the data so that the distance between the means decreases as *J*_*q*_ increases and to set $\sigma_{q}^{2}$ such that *σ*_*q*_ decreases as *J*_*q*_ increases. Denote $M_{J_{q}}=\left\{\mu_{q1}<...<\mu_{qJ_{q}}\right\}$. The value of *J*_*q*_ is allowed to vary in different iterations; as *J*_*q*_ increases, the set $M_{J_{q}+1}$ is constructed by adding the median of one of the intervals [*μ*_*q*,*j*_, *μ*_*q*,*j*−1_]. More details on the choice of the mean sequence and the variance are presented by [19].

**Fig 1:**
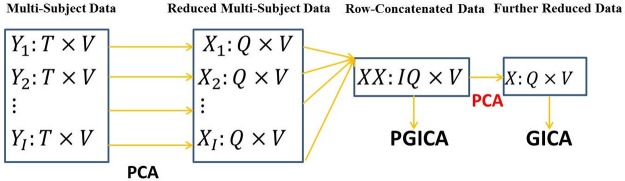
The work flow of PGICA. PCA is first applied to each subject for dimension reduction, then the dimension-reduced matrices are stacked together and PGICA is applied; as a comparison, a second dimension reduction step is usually required by fastICA algorithm. PGICA can potentially be used in combination with any of the existing back reconstruction methods such as dual regression and GIG-ICA.

Since the underlying independent components are the same for all subjects, the length of the vector **S**(*q*, ⋅) depends only on the number of non-background voxels. In most fMRI studies **S**(*q*, ⋅) has a large sample size (≈70,000 voxels for example), hence nonparametric estimation of the density can be problematic. To address this issue, [14] proposed a binning algorithm for the density estimation, essentially looking at the approximation to the histogram of the data. With this binning procedure, the weights of the mixture densities in Eq (3) given by (*θ*_*q*1_, …, *θ*_*qJ*_*q*__) are estimated using a constrained EM algorithm. The resulting density estimates satisfy the moment constraints required for full identifiability of the model by *E*[**S**(*q*, ⋅)] = 0, *E*[**S**(*q*, ⋅)^2^] = 1, 0 < *E*[**S**(1, ⋅)^3^] < … < *E*[**S**(*Q*, ⋅)^3^], for *q* = 1, ⋯, *Q*. Given the density estimation above as ${\hat{f}}_{1},\cdots ,{\hat{f}}_{Q}$, the log likelihood function of matrix **W** can be constructed as

$$\begin{array}{c} L(W,\hat{f})=\sum_{i=1}^{I}\{\sum_{v=1}^{V}\sum_{q=1}^{Q}[{\hat{f}}_{q}(\sum_{l=1}^{Q}w_{iql}x_{ilv})]+V\text{log}\left|\text{det}W_{i}\right|\}, \end{array}$$ (4)

where ${\hat{f}}_{q}\left(s\right)=\sum_{j=1}^{J_{q}}{\hat{\theta }}_{qj}\frac{1}{\sigma_{q}}\phi (\frac{s-\mu_{qj}}{\sigma_{q}}).$ The maximum of Eq (4) can be found by Quasi-Newton algorithm. The algorithm proceeds by iterating between the estimation of $\hat{f}$ and **W** until convergence. The complete algorithm pseudo code for fitting PGICA is given below and the work flow of PGICA is shown in Fig 1.

### PGICA

For each iteration *M*

1. Let $S_{i}^{\left(M\right)}=W_{i}^{\left(M-1\right)}X_{i}$, for each *i* = 1, …, *I*.
2. For each Independent Component q construct the set of midpoints *M*_*q*1_, …, *M*_*qp*_. of the bins and the corresponding counts *c*_*q*1_, *c*_*q*2_, …, *c*_*qp*_.
3. For each *q* = 1, …, *Q*, construct the set of means $M_{J_{q}^{\left(M\right)}}⊃M_{{J_{q}}^{\left(M-1\right)}}$ and the variance component *σ*_*q*_.
4. Estimate $\left(\theta_{q1}^{\left(M\right)},\ldots ,\theta_{qJ_{q}^{\left(M\right)}}^{\left(M\right)}\right)$ using MDE.
5. For each *i* = 1, …, *I*, compute the gradient $L^{\prime }\left({\hat{W_{i}}}^{\left(M\right)}\right)$ and hessian matrix $L^{\prime \prime }\left({\hat{W_{i}}}^{\left(M\right)}\right)$ **in parallel**.
6. For each *i* = 1, …, *I*, update the unmixing matrix ${\hat{W_{i}}}^{\left(M+1\right)}={\hat{W_{i}}}^{M}-L^{\prime \prime }{\left({\hat{W_{i}}}^{\left(M\right)}\right)}^{-1}L^{\prime }\left({\hat{W_{i}}}^{\left(M\right)}\right)$.
7. $\delta =\text{max}\;|{\hat{W_{i}}}^{\left(M+1\right)}-{\hat{W_{i}}}^{M}|.\;\text{If}\;\delta >\epsilon$ return to step 1.

In each iteration, the average of all **S**_*i*_ matrices are taken as **S** to estimate source density function. In this algorithm, Step 5 is the most time-consuming. Fortunately, the structure of the likelihood in Eq (4) makes it possible to simplify computations. Note that the likelihood is a product of the likelihoods of multiple subjects. Thus after taking logs, the gradients for different *W*_*i*_ s do not depend on each other. As a consequence, one can calculate the gradients and Hessians in parallel. According to Amdahl’s law [20], the theoretical speedup obtainable using parallelization is $\text{speedup}=\frac{1}{\frac{P}{N}+S}$, where *P* is the parallel proportion of the computations, *S* is serial proportion of the computations and *N* is the number of processors. Here *P* and *S* differ when the sizes of input data differ. The parallel proportion increases with the number of subjects. It encompasses more than 90% of the theoretical time for 300 or more subjects. Of course, the practical speedup will not be exactly the same as the theoretical one due to many factors such as messages passing overhead; see Section 5 for more information.

## 3 Simulation set-ups and data description

### 3.1 Simulation set-ups

**Fig 2:**
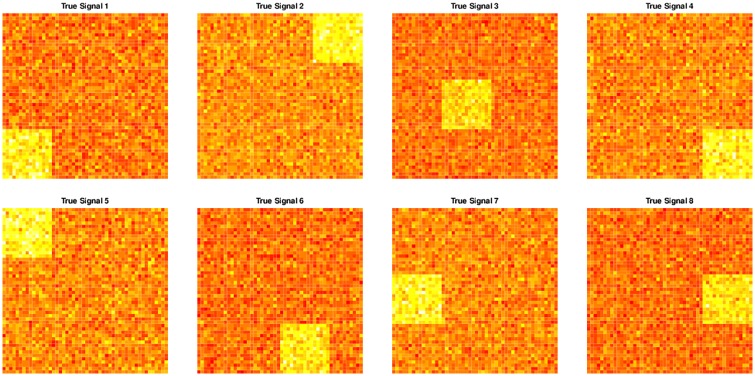
True signals for the simulation examples. Each component is a two dimensional array where the pixels in a square have higher intensities than the rest of the array. A random noise is added to each of the components at all pixels.

To demonstrate the validity of the proposed method and compare the accuracy of the parameter estimates with the commonly used fastICA algorithm we considered simulated data using two different simulation scenarios. We considered various shapes in the underlying independent components to estimate the accuracy of prediction of the brain networks in the imaging context. The first four shapes shown in Fig 2 are used in the first scenario, while all 8 underlying signals are used for the second simulation. The fastICA method [21] is used as a comparison. The mixing matrices for each subject are predefined in each simulation example. The underlying sources are generated for 100 simulation runs as described below. The observed matrices for each subject are then computed and fastICA and PGICA are used to estimate the mixing matrices for each subject and the underlying sources. Finally, the correlations of each component with the true underlying sources are calculated. Ideally, these correlations should be equal to 1 if the networks are perfectly estimated. For each example, we averaged the correlations for all the underlying components for each of the simulation runs and presented the boxplots of logarithms of the correlations for better visualization for each method in each simulation scenario to compare the results. The goal of the simulation studies is to compare the parameter estimates in high dimensional settings and demonstrate the performance of the proposed method in estimating the parameters. The real data examples show the power of the proposed method to perform group ICA in settings where other algorithms would fail because of the dimensionality of the data.

*Simulation 1:* Suppose there are 4 subjects and 4 underlying sources, i.e. *I* = 4 and *Q* = 4. Only 4 subjects are included in this simulation study so that all model generating parameters can be included in the paper for reproducibility. The data are generated from the group ICA model **X**_**i**_ = **A**_**i**_**S**, with *T* = *Q* = 4 and *V* = 2500 where the independent components are the first 4 signals in Fig 2. The four mixing matrices are defined as follows.

$$\begin{array}{c} A_{1}=(\begin{array}{cccc} 2 & 1 & 2 & 3\\ 3 & 3 & 1 & .5\\ 1 & 2 & 2 & 4\\ 4 & 3 & 2 & 1 \end{array}),A_{2}=(\begin{array}{cccc} 2 & 3 & 2 & 1\\ 3 & 4 & 1 & .5\\ 3 & 2 & 3 & 4\\ 2 & 3 & 3 & 1 \end{array}) \end{array}$$

$$\begin{array}{c} A_{3}=(\begin{array}{cccc} 1 & 2 & 2 & 1\\ 3 & 4 & 1 & .5\\ 3 & -1 & 3 & 4\\ 2 & 1 & 3 & 1 \end{array}),A_{4}=(\begin{array}{cccc} 3 & 2 & 2 & -1\\ 3 & 3 & 2 & 1\\ 3 & 1 & 1 & 4\\ 1 & 1 & 4 & .5 \end{array}). \end{array}$$

*Simulation 2:* In this example, we assume that the number of subjects is 50 while the number of underlying components is 8, *I* = 50, *Q* = 8. The data are, again, generated from the group ICA model **X**_**i**_ = **A**_**i**_**S**, with *T* = *Q* = 8 and *V* = 2500 where the independent components are the signals in Fig 2. Here, the components #4 and #6 were generated such that the “activated” regions in the two components are spatially overlapping, while the signals are still statistically independent.

The above simplified simulations demonstrate the estimation accuracy of PGICA algorithm compared with other commonly used estimation methods. In a more complex simulation experiment we simulate the variability of individual components and additional noises to examine the robustness of the algorithm. The SimTB toolbox in MATLAB is a very convenient tool for such analysis. Simulation 3 is performed using SimTB to test the algorithm’s performance with different individual components and noise vectors.

**Fig 3:**
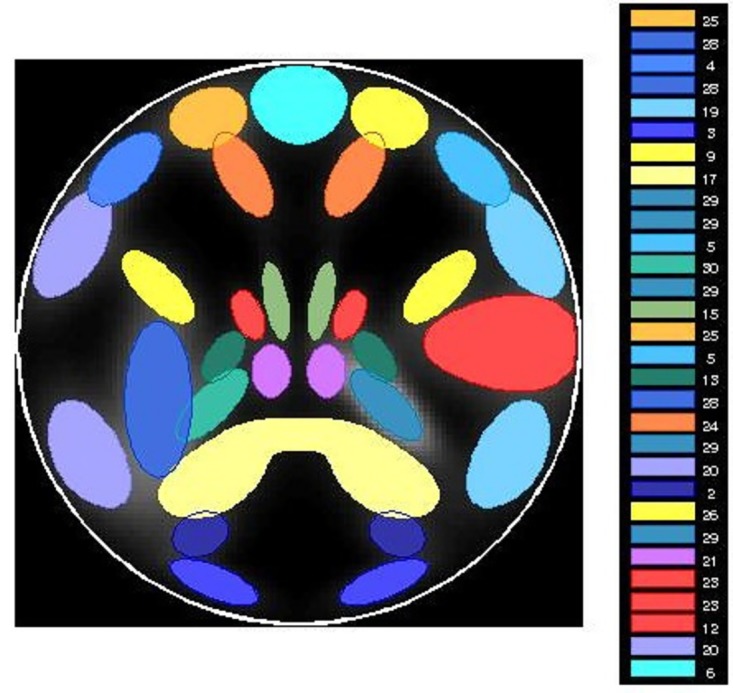
Map of available source in simulation 3.

*Simulation 3:* In this simulation, *N* = 30 experiments were performed. In each experiment, three methods (fastICA, InfoMax ICA and PGICA) are applied to analyze the simulation data. The accuracies of estimated independent components from all three methods are compared using a paired t-test. The simulation data for each experiment is generated with the SimTB toolbox. The simulation configurations are: in each experiment, *nC* = 10 components are randomly chosen from the 30 available sources; *M* = 5 subjects; *nV* × *nV* = 2500 voxels; *nT* = 100 time points; a Rician noise is always added. The same accuracy measure is used as in the above two simulations. A map of the 30 underlying sources are shown in Fig 3. Once again, some of the sources were designed to spatially overlap while their signals are still statistically independent.

### 3.2 1,000 functional connectomes project data

First, PGICA was applied to data from the 1,000 Functional Connectomes Project, which consists of thousands of resting state scans combined across multiple sites with the goal of facilitating discovery and analysis of brain networks [22]. The quality and scanning parameters vary across sites. Thus, we focus on data from two sites that each provided a large number of scans: Cambridge and Oulu. We include 301 subjects in the analyses presented below, 198 are from Cambridge and 103 from Oulu. As discussed above, directly applying currently used group ICA methods to data of this size is computationally infeasible for regular computers due to limitations of memory and running time. As such, it provides an important test case for PGICA.

Scanning parameters used to acquire the data from each site are detailed elsewhere (for complete information see http://fcon_1000.projects.nitrc.org/fcpClassic/FcpTable.html). Each subject’s data consisted of either 119 time points collected every 3 s or 245 time points collected every 1.8 s. Note that even though the number of time points varies across subjects, the algorithm can still be applied, as the first PCA step reduces the dimensions of each dataset to be the same. However, the variance related consequences of including data with varying scan lengths and sampling frequencies remain an open topic. All scans were collected using a 3T scanner. The data were preprocessed using the processing scripts available on the NITRC website (www.nitrc.org/projects/fcon_1000/). Anatomical images were de-obliqued, reoriented, and skull stripped, while the functional scans were de-obliqued, reoriented, motion corrected, skull stripped, grand mean scaled, temporal bandpass filtered, and de-trended (linear and quadratic). Functional scans were registered to anatomical scans using FLIRT in FSL [23]. The structural scans were registered to the Montreal Neurological Institute (MNI) space using FLIRT and the transformation was subsequently applied to the functional scans. A mask based on the MNI template is used to separate the background of the images. For each time point, the 3D array is vectorized to obtain a V dimensional vector of intensities that are then concatenated over time. Hence we obtain a *T* × *V* dimensional matrix **X**_**i**_ for each subject. PCA is applied to each matrix and reduces the temporal dimension *T* to *Q* = 20. PGICA is then applied to these **X**_**i**_ matrices.

### 3.3 Autism brain imaging data exchange

Next, PGICA was applied to data from the Autism Brain Imaging Data Exchange (ABIDE) consortium, a collaboration between 17 imaging centers to openly share existing resting state fMRI scans with corresponding structural MRI and phenotypic information. In total, the database consists of 539 individuals with autism spectrum disorder (ASD) and 573 age-matched typical controls [24]. Site-specific protocols for recruitment and image acquisition are available online (http://fcon_1000.projects.nitrc.org/indi/abide); in short, 5 to 10 minutes of rs-fMRI data collected using repetition times (TR) between 1.5 s and 3 s were shared for each subject. The first 10 s of each resting state scan were ignored to allow for magnetization stabilization. Resting state scans were then slice-time adjusted using the slice acquired in the middle of the TR, and rigid body realignment parameters were estimated with respect to the first (stabilized) functional volume. An iterative process previously described by [25] was used to coregister and normalize the structural and functional images to MNI space. Each resting state scan was then temporally detrended on a voxel-wise basis and spatially smoothed (2-mm FWHM Gaussian kernel). Finally, each resting state scan was downsampled by randomly sampling 67,749 of the 229,263 non-background voxels to reduce computation demands. Downsampling the voxels is only performed to estimate starting values of the parameters for initialization of the algorithm, but is not necessary for the algorithm itself. The FSL package was used to smooth the original NIFTI images [23].

**Fig 4:**
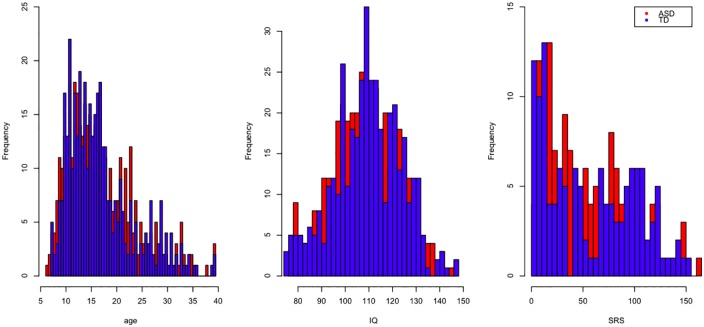
Histograms of age (left), IQ (middle), and SRS (right) for participants in ABIDE plotted and colored by disease diagnosis and overlaid, where blue corresponds to typically developed (TD) controls and red corresponds to ASD individuals.

As opposed to the first application presented in this paper, we found that a much larger subset of the data can be used for simultaneous analysis due to the data quality and consistency across the sites. Because they made up a low percentage of the total number of subjects (∼10%), girls were excluded from the analysis. Age was restricted to individuals between 6 and 40 years old. Individuals with framewise displacement more than two standard deviations away from the mean were also excluded from the analysis. The data collected at the Kennedy Krieger site was also excluded from the analysis for comparison of the results in future studies. As a result, scans for 779 subjects are analyzed in this application, 400 typical controls, 379 individuals with ASD. The histograms of age, the intelligence quotient (IQ), and the social responsiveness scores (SRS) are shown in Fig 4.

## 4 Results

### 4.1 Simulation result

**Fig 5:**
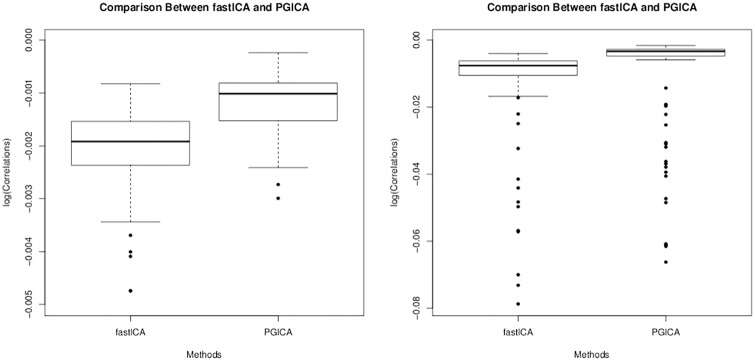
Boxplots (for both fastICA and PGICA) of the average correlations (log-transformed) of the true signals with the estimated signals from simulation 1 on the left and simulation 2 on the right.

*Simulation 1 Result:* The boxplots of the average correlations across four independent components for each of the 100 simulation runs are shown in Fig 5 while the summary statistics of the estimated correlations are presented in Table 1. The two methods perform similarly to each other with PGICA performing marginally better than fastICA.

**Table 1:** Summary measures of the correlations in the two simulation examples.

		min	1st quantile	median	3rd quantile	max
Sim 1	fastICA	0.9953	0.9977	0.9981	0.9985	0.9992
	PGICA	0.9944	0.9985	0.9990	0.9992	0.9998
Sim 2	fastICA	0.9243	0.9895	0.9924	0.9938	0.9960
	PGICA	0.9359	0.9952	0.9966	0.9972	0.9984

*Simulation 2 Result:* The results of the 100 simulation examples shown in Fig 5 and Table 1 demonstrate that the correlations of the estimated components with the true underlying signals using the proposed PGICA method are significantly better than those estimated using the conventional fastICA algorithm.

*Simulation 3 Result:* The comparison of three ICA algorithm is summarized in Table 2. A paired t-test was used to compare the performance of PGICA and InfoMax, the difference is 0.01 with a p-value of 1.6*e*^−6^. Similarly, comparison between PGICA and fastICA has difference of 0.01 and p-value of 1.8*e*^−6^. This simulation shows that PGICA gives more accurate estimation accuracies with varying underlying sources and noises. The improvement in accuracy when using PGICA is small relative to both Infomax and fastICA, but statistically significant.

**Table 2:** Compare fastICA/InfoMax ICA/PGICA accuracies.

	min	1st quantile	median	mean	3rd quantile	max
fastICA	0.5713	0.7378	0.7809	0.7901	0.8761	0.9802
InfoMax	0.5699	0.7387	0.7814	0.7903	0.8764	0.9802
PGICA	0.5730	0.7474	0.8003	0.8008	0.8879	0.9840

### 4.2 1,000 functional connectomes project result

**Fig 6:**
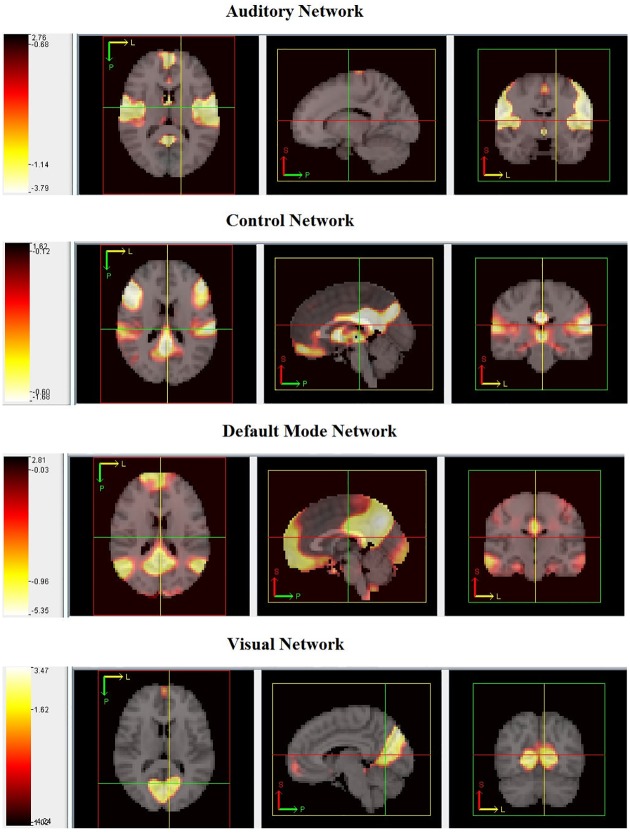
Axial, sagittal, and frontal (left to right) planes of the auditory, control, default mode and visual networks (from top) estimated using 301 fMRI scans from the 1,000 Functional Connectomes Project dataset. The thresholded maps are overlaid on a greyscale MNI template brain. The 90th slice is shown from the MNI template in each of the plots. The colors correspond to the intensities in the estimated brain networks where white: high intensity to red: low intensities.

**Fig 7:**
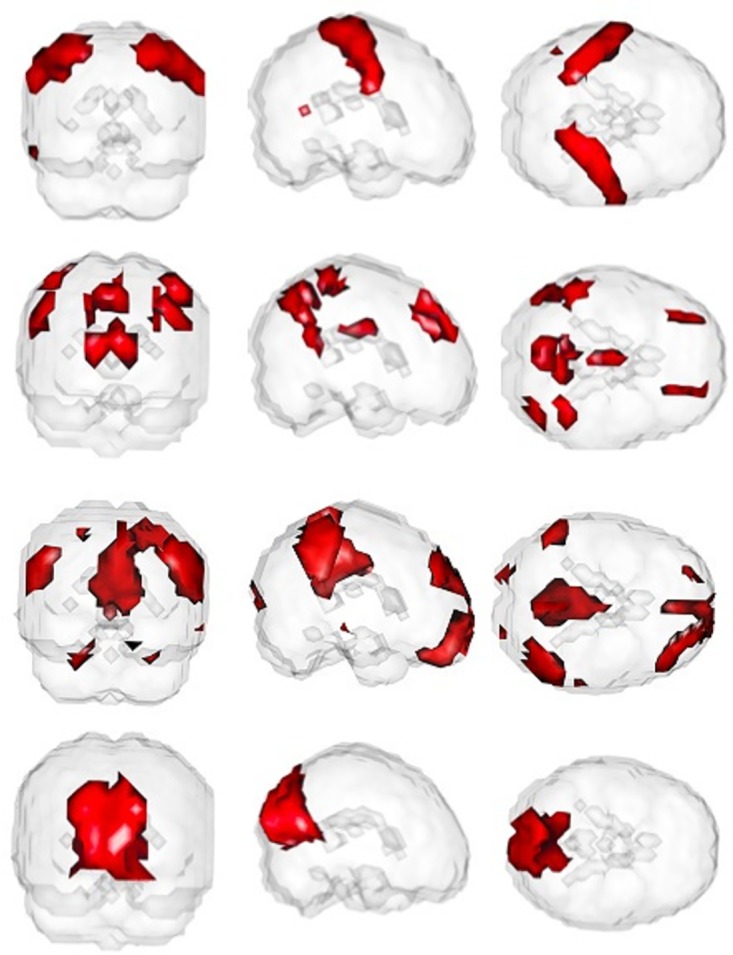
3D view of auditory, control, default mode and visual networks (from top).

Following the design of group ICA analysis described by [22], group ICA was used to obtain *Q* = 20 components for the 301 subjects in the 1,000 Functional Connectomes Project Dataset. Fig 6 shows axial, sagittal, and frontal planes of four of the estimated networks by PGICA: auditory, control, default mode, and visual. The estimated networks are thresholded at (5%) and the map is overlaid on a grayscale template MNI image. The networks shown in this example were identified visually as a proof of concept exercise. 10 out of the 20 networks are identified as noise-related. The estimated networks have clear edges and less noise in the areas that are not a part of the networks showing the importance of estimating the networks using larger datasets. Fig 7 shows three dimensional renderings of the same networks shown in Fig 6 colored in red and overlaid on an opaque template image confirming that the estimated networks are more noise-free.

The increase in speed when using PGICA as compared to non-parallel version of the algorithm called HDICA (high dimensional ICA) as the number of subjects increases is shown in Table 3. The memory usage of HDICA increases linearly with the number of subjects (memory usage = number of subjects × single subject), while the memory usage of PGICA remains constant as the number of subjects increases (memory usage = single subject). For PGICA, each slave computer only calculates the gradient and Hessian for a single subject, as long as we have enough slave computers. In practice, total memory usage is several times higher than just the input data. Thus memory usage of HDICA quickly goes beyond the ability of even super computers, making it incapable of dealing with large groups of observations.

**Table 3:** Speed increase of PGICA.

# of subjects	1	10	50	150	300
Non-Parallel GICA time (min)	20	400	4000	12000	NA
PGICA time (min)	20	80	592	2000	4100

In this example, 15 computing clusters were used for estimation using the PGICA on a Sun Grid Engine (SGE). All computations are performed on clusters with the same or very similar hardware properties such as speed and age.

### 4.3 Autism brain imaging data exchange result

**Fig 8:**
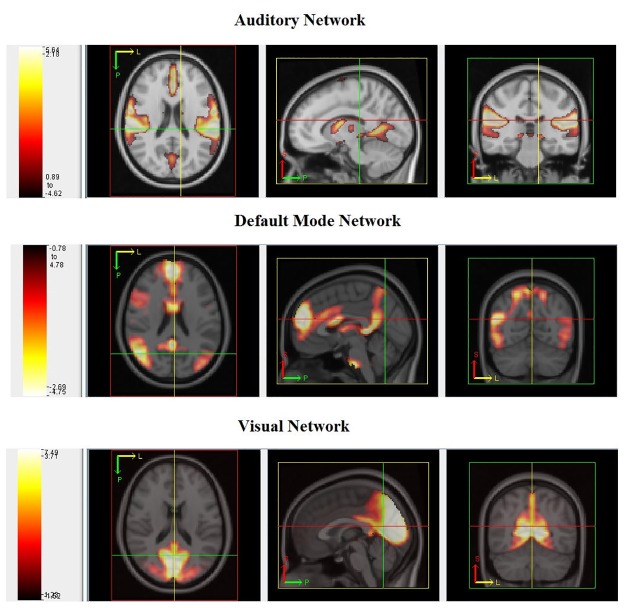
Axial, sagittal, and frontal (left to right) planes of the default mode, auditory and visual networks (from top) estimated using 779 fMRI scans from the ABIDE dataset. The thresholded maps are overlaid on a greyscale MNI template brain. The colors correspond to the intensities in the estimated brain networks where white: high intensity to red: low intensities.

Similar to the data analysis performed for the 1000 FCP data in Section 4.2, we estimated *Q* = 20 components using the fMRI scans for 779 participants in the ABIDE sample. We used a semi-automated method to classify estimated ICs as signal or noise and to assign functional labels to signal components. First, we calculated the spatial correlation between each of our 20 group-level ICs and a publicly available set of 75 ICs that were estimated from resting state data collected from healthy adults and have already been classified as resting state networks or noise by a group of experts [26]. We then manually inspected all ICs to ensure that they were correctly classified. Using this two-stage method, we classified 11 ICs as noise and 9 as signal. Examples of the 9 signal components are shown in Fig 8. The clear edges of the estimated signal components further demonstrate the ability of the proposed method to estimate ICs for such high dimensional data.

This example is one of the few direct runs of group ICA for rs-fMRI in the literature for such high dimensional data. One of the largest group ICA runs we identified in the literature is presented by [26] and is based on rs-fMRI data for 603 healthy adolescents and adults. Before [26] could run group ICA, they first had to reduced their data to 75 principal components using the expectation-maximization algorithm included in GIFT to avoid “otherwise prohibitive memory requirements”. The uniqueness of the proposed algorithm is the application to the whole dataset directly which can provide new insights for group comparisons without the necessity of splitting the groups into parts. In addition, the algorithm can be applied to data with even larger numbers of subjects barring any issues with data quality.

## 5 Discussion

In this paper, we extended the group ICA algorithm of [14] using high-performance computing. The new PGICA algorithm can analyze large-scale data efficiently. Essentially, the sequential nature of the algorithm turns a memory-intensive, constant-time computing problem into a constant-memory, time-intensive problem, and then uses parallel computing to turn the resulting time-intensive problem into a constant time problem. With this algorithm, two large resting-state fMRI datasets were analyzed. An interesting byproduct of this work is a comprehensive brain network atlas from over 300 healthy adults and another based on 779 scans which include both ASD individuals and control subjects.

Although the method of [14] is theoretically scalable in terms of its memory requirements, the approach requires the serial calculation of gradients to optimize parameter estimation, which can be very slow for high-dimensional data to the point where it would still be practically infeasible in terms of computation time. Computing the gradients for different subjects in parallel could potentially speed up the algorithm dramatically, provided the cost is much lower than the necessary data transfer time. Parallel programming has been widely utilized in scientific computing since the 1950s [27]. According to Flynn’s taxonomy [28], most current computers are Multiple Instruction Multiple Data (MIMD) systems. MIMD computers are typically categorized as shared-memory, distributed-memory or hybrid systems. In a shared-memory system, all processes share addressable memory and communicate via shared variables. In distributed-memory systems, such as supercomputers and clusters, one process communicate with others through message passing. A supercomputer’s processors and the network infrastructure are tightly coupled and specialized for parallelization. In contrast, clusters are composed of off-the-shelf computers connected by an off-the-shelf network. Recently, General-purpose computing on graphics processing units, or GPGPU, is developing fast and provides a new scheme for parallel computing. In this work, the PGICA algorithm is performed on both shared-memory and distributed-memory systems. It can be implemented to fit other parallel computing schemes in the future. One limitation of the proposed algorithm is that its performance depends on the available parallel computing capacity. The running speed will be limited if one doesn’t have the required computing power. As a comparison, commonly used ICA methods which require a group-level dimension reduction preprocessing step are mainly limited by memory size. As the memory requirement is quadratic, it will run out quickly as the data grow.

We used an extensive simulation study to validate the accuracy of the proposed algorithm in high dimensional settings. The simulation studies show that the proposed PGICA algorithm performs as well as commonly used methods. Using the measure of correlation between estimated and true signal, it is performing better than the compared methods (the outperformance is small, but statistically significant). The information provided on the computation time gain is presented using the real data example as the purpose of the simulation studies was to assess accuracy rather than required computation time. In principle, given enough nodes the algorithm can be applied to a dataset with any number of subjects and the simulation results indicate that the accuracy of the results will improve with the increased number of subjects under the assumption of no biologically irrelevant systematic differences between subgroups in the data.

Large, freely available multisite datasets such as the 1000 FCP and ABIDE are invaluable for a number of reasons including accelerating neuroimaging discovery science and providing a means to validate neuroimaging findings through replication. However, these datasets also contain some inherent limitations. Each participating data collection site was motivated by its own research questions, leading to potentially large inconsistencies in acquisition parameters, subject populations, and research protocols across sites that may limit our ability to estimate networks and our sensitivity to detect biologically meaningful group differences. We did not analyze the 1000 Functional Connectome Project dataset in its entirety, as there are site-specific variations, which plague the quality of results. In this paper, we focused on two sites to minimize the influence of site-specific effects. The work presented in this paper shows that estimating networks using data from a large number of subjects can result in highly precise estimates of the networks. However, if the variability between the scans for the subjects in the data is very high (especially due to biologically unrelated reasons), it can obscure the results instead of improving the estimates. Developing statistically principled approaches to removing technical variability from resting state fMRI data collected from multiple sites is an important avenue of future work.

The functional imaging scans in the ABIDE dataset, while still collected in various data collection sites, was more homogeneous when analyzing the data together. We analyzed a subset of 779 fMRI scans simultaneously in this paper. The networks identified in this example can be used as a powerful tool for exploring possible differences in network engagement over time between the two groups: ASD and TD, using the second level analyses as described by [29]. The ICs we estimated from the ABIDE assume common spatial maps for all subjects in the study including those with ASD and their TD peers. A question still remains whether the spatial networks are the same between the two groups or whether the proposed method can be used to test the hypothesis of significant differences between spatial networks of each group. In this example, we used a downsampling approach to estimate the starting values of the parameters for our model. While not implicitly stated by the proposed method, the voxel intensities in the observed images are assumed to be statistically independent. The assumption may be violated when the voxel sizes are very small and the correlations between neighboring voxels may not be small enough to be ignored. Hence, as the spatial resolution of images improves a more thorough investigation of the effect of spatial correlations on the parameter estimates will be necessary. It is interesting to note that the regions comprising networks defined using ABIDE are generally more diffuse than those defined using the 1000 FCP set. This can be seen more strikingly for the DMN and visual networks, which could suggest differences between ASD and TD children. The networks identified using the proposed method can be used to investigate these questions further.
